# Haematological and fibrinolytic status of Nigerian women with post-partum haemorrhage

**DOI:** 10.1186/s12884-018-1794-1

**Published:** 2018-05-09

**Authors:** Ian Roberts, Haleema Shakur, Bukola Fawole, Modupe Kuti, Oladapo Olayemi, Adenike Bello, Olayinka Ogunbode, Taiwo Kotila, Chris O. Aimakhu, Tolulase Olutogun, Beverley J. Hunt, Sumaya Huque

**Affiliations:** 1 Clinical Trials Unit, London School of Hygiene and Tropical Medicine, Keppel Street, London, WC1E 7HT UK; 2 Department of Obstetrics & Gynaecology, National Institute of Maternal and Child Health, College of Medicine, University of Ibadan, Orita-Mefa, Ibadan, Nigeria; 3 Department of Chemical Pathology, College of Medicine, University of Ibadan, Orita-Mefa, Ibadan, Nigeria; 4 Department of Obstetrics & Gynaecology, College of Medicine, University of Ibadan, Orita-Mefa, Ibadan, Nigeria; 5 Department of Haematology, College of Medicine, University of Ibadan, Orita-Mefa, Ibadan, Nigeria; 6 Thrombosis & Haemophilia Centre, Guy’s & St Thomas’ Trust, St Thomas’ Hospital, Lambeth Palace Road, London, UK

**Keywords:** Fibrinolysis, Coagulation, Postpartum haemorrhage

## Abstract

**Background:**

Early treatment with tranexamic acid reduces deaths due to bleeding after post-partum haemorrhage. We report the prevalence of haematological, coagulation and fibrinolytic abnormalities in Nigerian women with postpartum haemorrhage.

**Methods:**

We performed a secondary analysis of the WOMAN trial to assess laboratory data and rotational thromboelastometry (ROTEM) parameters in 167 women with postpartum haemorrhage treated at University College Hospital, Ibadan, Nigeria. We defined hyper-fibrinolysis as EXTEM maximum lysis (ML) > 15% on ROTEM. We defined coagulopathy as EXTEM clot amplitude at 5 min (A5) < 40 mm or prothrombin ratio > 1.5.

**Results:**

Among the study cohort, 53 (40%) women had severe anaemia (haemoglobin< 70 g/L) and 17 (13%) women had severe thrombocytopenia (platelet count < 50 × 10^9^/L). Thirty-five women (23%) had ROTEM evidence of hyper-fibrinolysis. Based on prothrombin ratio criteria, 16 (12%) had coagulopathy. Based on EXTEM A5 criteria, 49 (34%) had coagulopathy.

**Conclusion:**

Our findings suggest that, based on a convenience sample of women from a large teaching hospital in Nigeria, hyper-fibrinolysis may commonly occur in postpartum haemorrhage. Further mechanistic studies are needed to examine hyper-fibrinolysis associated with postpartum haemorrhage. Findings from such studies may optimize treatment approaches for postpartum haemorrhage.

**Trial registration:**

The Woman trial was registered: NCT00872469; ISRCTN76912190 (Registration date: 22/03/2012).

## Background

Tranexamic acid (TXA) administration reduces death due to bleeding in trauma patients. Among patients treated within 3 h of injury, TXA reduces death due to bleeding by one third [1–3]. Early activation of fibrinolysis is common after serious injury and contributes to the coagulation abnormalities seen in bleeding trauma patients [4, 5]. Hypo-perfusion and tissue injury are thought to start the coagulopathy, although we do not understand the molecular pathways [6].

Results from the WOMAN trial, a large randomised trial conducted primarily in low and middle income countries, show that early tranexamic acid use reduces death due to bleeding after postpartum haemorrhage (PPH) [7]. This suggests that fibrinolysis is also an important pathophysiological mechanism in obstetric bleeding. However, whereas increased fibrinolysis is common in trauma, its association with PPH is less well known [8]. We report the prevalence of hyper-fibrinolysis in women with PPH in Nigeria.

## Methods

The WOMAN trial was a randomised placebo controlled trial of the effect of tranexamic acid on death, hysterectomy and other surgical interventions in women with clinically diagnosed primary PPH [7]. Although the diagnosis was clinical, we specified that diagnosis of primary PPH could be based on an estimated blood loss of more than 500 mL after vaginal birth or 1000 mL after caesarean section, or any blood loss sufficient to compromise haemodynamic stability. The diagnosis of PPH could also be made using clinical judgement, independent of the volume of blood loss. We have described the methods in detail elsewhere [7]. We examined haematological (full blood count) and haemostatic parameters in a sample of 167 trial participants recruited at University College Hospital, Ibadan, Nigeria. A total of 309 women were recruited into the WOMAN trial at University College Hospital. Because of occasional equipment failures and lack of reagents the 167 (54%) women included in the ETAC study were not a consecutive series. Although most of the recruited women delivered at University College Hospital, some patients were transferred from outlying health facilities after they had developed PPH because they required urgent medical support. In these women, blood loss was estimated based on the history and observed blood loss.

After completing consent procedures but before giving the trial treatment (TXA or placebo), we drew approximately 15 mL of venous blood and divided the sample into three vacutainer tubes. We collected one 5 mL sample in a tube containing potassium EDTA for full blood count analysis and two 4.5 mL samples in tubes containing 0.5 mL sodium citrate (0.109 mol/L) for coagulation and rotational thromboelastometry. We used a five-parameter particle counter Sysmex KN analyser (Sysmex Corporation, Kobe, Japan) for the blood count analysis. Anaemia was defined according to the World Health Organisation definition of anaemia in pregnancy as a haemoglobin below 110 g/L and below 70 g/L for severe anaemia [9].

**Fig. 1:**
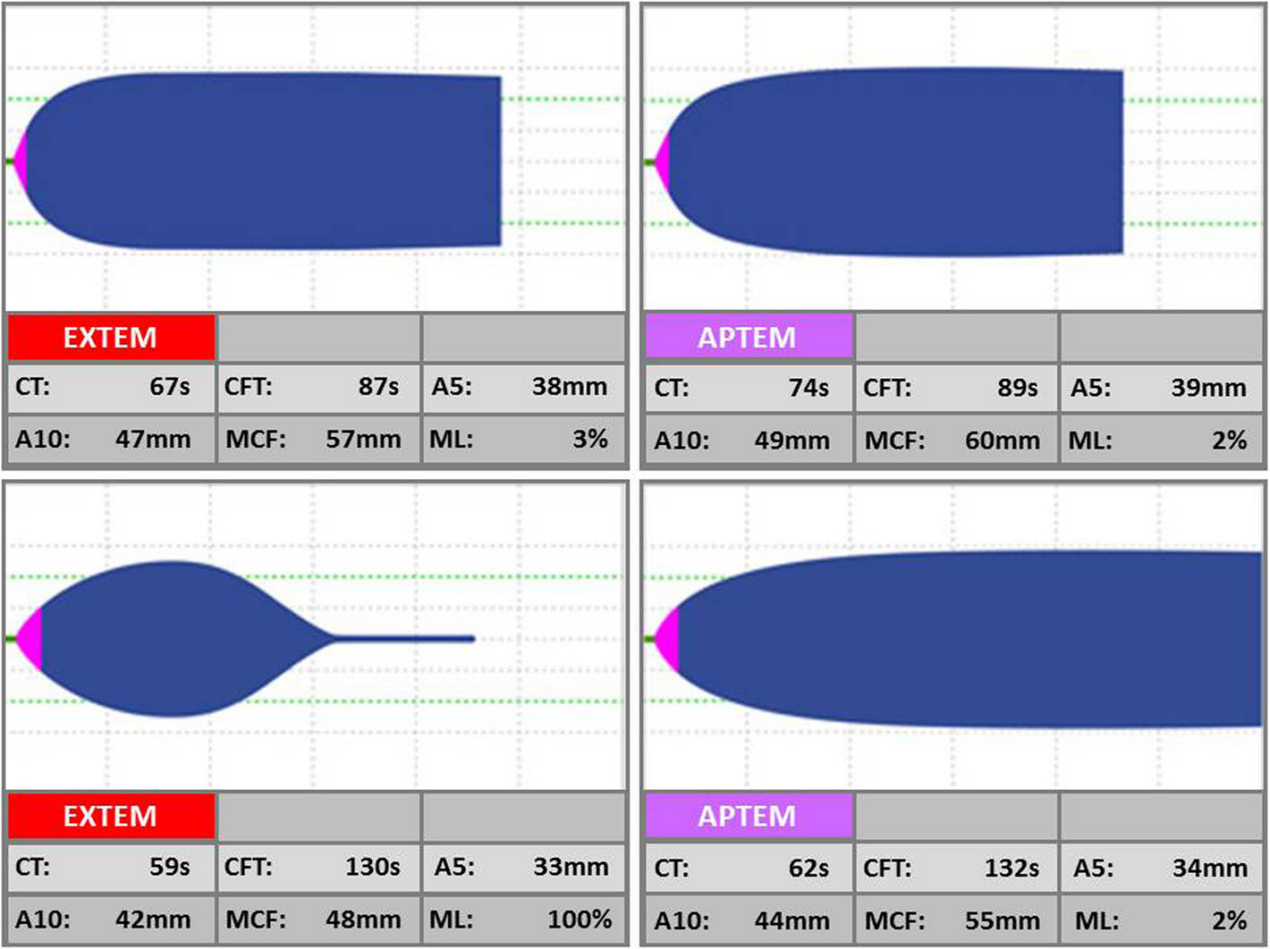
ROTEM traces with (top) and without (bottom) hyper-fibrinolysis

**Fig. 2:**
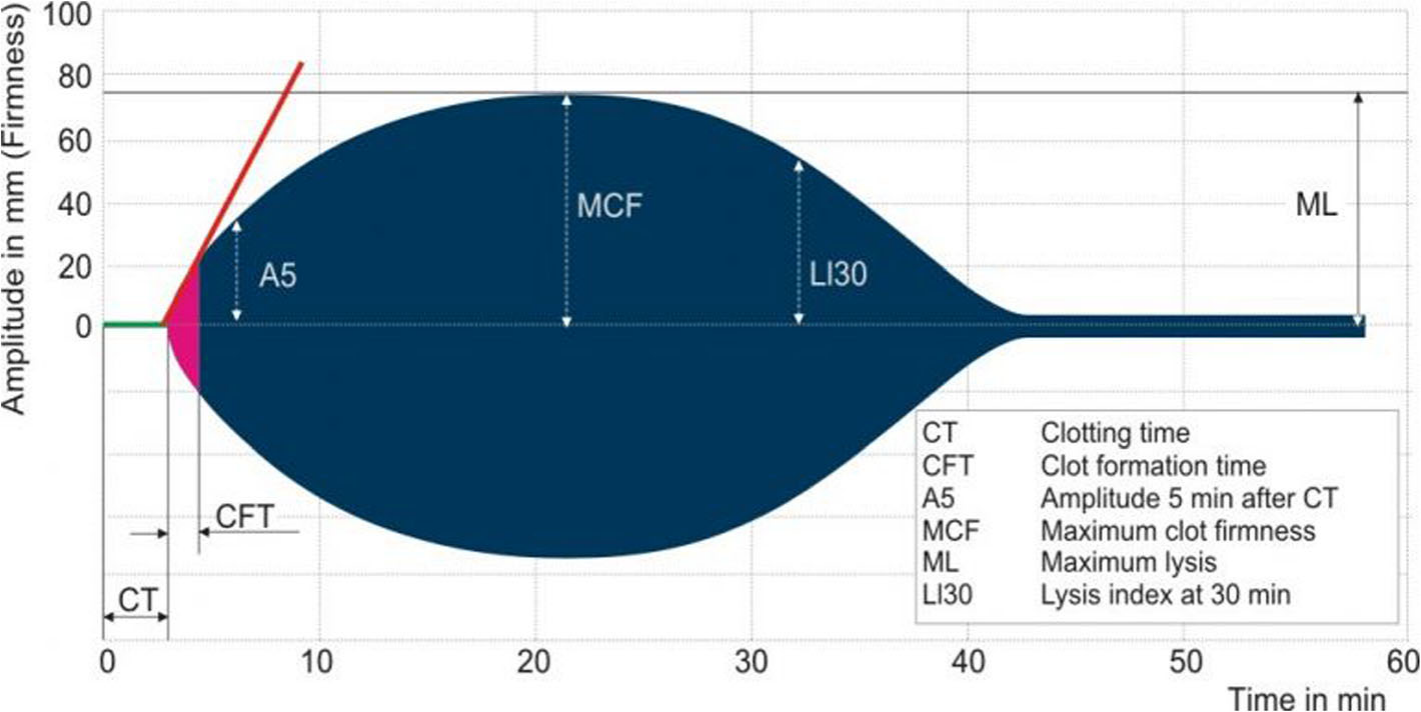
Annotated trace showing ROTEM parameters

After centrifuging the blood at 3000 g for 20 min, we measured prothrombin time (PT), normalised prothrombin ratio, activated partial thromboplastin time (aPTT), Clauss fibrinogen and D-dimers using a HumaClot Junior automated coagulation analyser. We measured ROTEM thromboelastometry parameters at 37 °C using two of the four channels (EXTEM, APTEM) of the ROTEM coagulation analyser [TEM®, Munich, Germany]). In EXTEM, coagulation is initiated using a small amount of tissue factor and the development of the clot is expressed in numbers and as a trace. In APTEM, coagulation is initiated in the same way, but the addition of aprotinin or tranexamic acid in the reagent inhibits fibrinolysis in vitro. By comparing the two traces, the extent of fibrinolysis can be assessed. If the sample is hyper-fibrinolytic, the same degree of clot lysis seen in EXTEM is not present in APTEM (Fig. 1). The following ROTEM variables were examined from the EXTEM and APTEM traces (Fig. 2): Clotting time (CT) which corresponds to the time required to trigger the process of coagulation, amplitude of the clot at 5 min and 10 min (CA5 and CA10, respectively), maximum clot firmness (MCF), clot lysis at 30 min and 60 min (LI30 and LI60, respectively), and maximum lysis (ML). We stored the ROTEM reagents at 2–8 °C with temperature monitoring and we used in date reagents.

We conducted quality control (QC) analyses as per the manufacturer’s recommendations. Before starting the study, TEM staff trained the Nigerian study team to use the ROTEM machine. We stored the ROTEM data on the machine with a backup after each analysis. We collected, cleaned and analysed the study data at the Clinical Trials Unit of the London School of Hygiene & Tropical Medicine. Fibrinolysis was assessed as the amount of clot lysis on EXTEM (https://www.rotem.de/en/methodology/interpretation/). We defined hyper-fibrinolysis as ML > 15% on ROTEM EXTEM. This definition of hyper-fibrinolysis is provided by the manufacturer and is widely used in research studies [4]. Coagulopathy was defined as an EXTEM A5 < 40 mm or a prothrombin ratio > 1.5. This A5 definition of coagulopathy was based on studies of the use of ROTEM to diagnose acute traumatic coagulopathy in which a A5 < 40 mm predicted the receipt of massive blood transfusion in 73% of patients [10, 11]. Normal ROTEM values for peri-partum Nigerian women have not been studied, so instead we have indicated the normal values from a study of 161 healthy peri-partum Dutch women [12].

## Results

Table 1 shows the characteristics of the 167 women. One hundred and twenty eight women (77%) gave birth in the hospital, whereas 39 (23%) gave birth in other settings and were admitted after PPH onset. Seventeen (10%) women received colloids during fluid resuscitation for PPH prior to sampling. The estimated mean blood loss at the time of randomisation and blood sampling was 1531 mL. One hundred and eight (65%) women lost more than 1000 mL of blood.

**Table 1:** Baseline characteristics of trial participants (*N* = 167)

	Count (%) / Mean(SD)
Age (years)	
*Mean (SD)*	31.9 (5.6)
*Range*	18–46
Time since delivery (hours)	
≤ 3	103 (61.7%)
> 3	64 (38.3%)
*Mean (SD)*	4.2 (5.4)
Type of delivery	
Vaginal	80 (47.9%)
Caesarean section	87 (52.1%)
Delivery in randomising hospital	
Yes	128 (76.7%)
No	39 (23.4%)
Cause of haemorrhage	
Uterine Atony	74 (44.3%)
Surgical trauma/tears	59 (35.3%)
Placenta praevia/accreta	21 (12.6%)
Other	12 (7.2%)
Unknown	1 (0.6%)
Systolic blood pressure (mm(Hg)	
< 90	31 (18.6%)
≥90	135 (80.8%)
Missing	1 (0.6%)
Mean (SD)	110 (27)
Estimated volume of blood loss (mL)	
≤ 1000	59 (35.3%)
> 1000	108 (64.7%)
*Median (25th, 75th percentile)*	1200 (1000, 2000)
*Range*	500–5530
Colloids given	
Yes	17 (10.2%)
No	150 (89.8%)

Table 2 shows the full blood count and haemostatic parameters. One hundred and sixteen (88%) women were anaemic (haemoglobin < 110 g/L) and 53 (40%) were severely anaemic (haemoglobin < 70 g/L) at the time of sampling. Thirty eight women (33%) had a microcytic picture (defined as an mean corpuscular volume < 80 fl), although there was no further investigation to discover if this was due to iron deficiency or thalassemia traits or both. Twenty-six women (21%) were lymphopenic (lymphocyte count < 1 × 10^9^/L). Forty-one (32%) women had thrombocytopenia (platelet count < 100 × 10^9^/L) and 17 (13%) had severe thrombocytopenia (platelet count< 50 × 10^9^/L).

**Table 2:** Haematological parameters of trial participants at baseline (*N* = 167)

	*n* ^a^	Mean (SD) / Count (%)	Median (25th, 75th centile range)	Normal range^b^
Full Blood Count Variables				
Red blood cell count (×10^12/L)	130	2.9 (1.0)	3.0 (2.3, 3.7)	3.8–5.8
Haemoglobin (g/L)	132	78.1 (35.6)	79.5 (56, 97)	110–165
Haemoglobin < 110 g/L		116 (87.9%)		
Microcytic (MCV < 80)		38 (32.8%)		
Normocytic (MCV 80–100)		75 (64.7%)		
Macrocytic (MCV > 100)		3 (2.6%)		
Haemoglobin < 70 g/L		53 (40.2%)		
Hematocrit (%)	127	24.2 (7.7)	24.7 (19.2, 30)	35–50
Hematocrit < 33%		109 (85.8%)		
Hematocrit < 25%		64 (50.4%)		
White cell count (×10^9/L)	131	11.9 (8.1)	11.2 (5.3, 16.9)	3.5–10.0
White cell count > 25 (×10^9/L)		8 (6.1%)		
Lymphocyte count (×10^9/L)	122	2.6 (2.4)	1.9 (1, 3)	1.2–3.2
Lymphocyte count < 1 (×10^9/L)		26 (21.3%)		
Differential white cell count (monocytes) (×10^9/L)	100	1.8 (1.8)	1.3 (0.6, 2.2)	0.3–0.8
Differential white cell count (granulocytes) (×10^9/L)	100	8.7 (5.6)	7.7 (5.1, 11.5)	1.2–6.8
Platelet count (×10^9/L)	130	155.2 (101.7)	137 (86, 208)	150–390
Platelet count < 100 (×10^9/L)		41 (31.5%)		
Platelet count < 50 (×10^9/L)		17 (13.1%)		
Coagulation variables				
Prothrombin time (PT, seconds)	137	19.0 (12.6)	15.2 (13.5, 18.8)	9.6–12.9
Normalised prothrombin ratio	133	1.2 (0.7)	1.0 (0.9, 1.2)	
Normalised prothrombin ratio > 1.2		29 (21.8%)		
Normalised prothrombin ratio > 1.5		16 (12.0%)		
Activated partial thromboplastin time (APTT, seconds)	133	35.9 (22.9)	30.4 (27.1, 35.6)	
Thrombin time (TT, seconds)	124	13.1 (12.5)	10.4 (9.1, 12.6)	
Normalised thrombin ratio	124	0.9 (0.9)	0.7 (0.6, 0.9)	
Fibrinogen (g/L)	136	8.4 (6.6)	6.6 (3.2, 12.3)	
Fibrinogen (< 1 g/L)		4 (2.9%)		
Fibrinogen (< 2 g/L)		16 (11.8%)		
Fibrinogen (< 4 g/L)		45 (33.1%)		
D-Dimer (mg/L)	119	8.6 (8.2)	5.7 (2.3, 13.5)	
Thromboelastometry (ROTEM® EXTEM) variables				
Clotting time (CT, seconds)	151	127.4 (336.9)	54 (45, 72)	34–66
Amplitude at 5 mins (A5, mm)	146	42.1 (15.8)	47 (35, 53)	
A5 < 40 mm		49 (33.6%)		
Amplitude at 10 mins (A10, mm)	149	51.6 (17.5)	58 (47, 63)	44–73
Maximum clot firmness (MCF, mm)	146	59.3 (17.5)	65 (56, 70)	55–78
Clot lysis at 30 min (LI30, %)	145	98.0 (10.8)	100 (100, 100)	
Clot lysis at 60 min (LI60, %)	101	93.3 (10.1)	96 (92, 99)	
Maximum Lysis (ML, %)	150	14.7 (20.4)	9 (4, 15)	0–44
ML > 15%		35 (23.3%)		
Thromboelastometry (ROTEM® APTEM) variables				
Clotting time (CT, seconds)	152	245.2 (882.3)	58 (46, 81)	31–71
Amplitude at 5 mins (A5, mm)	143	44.0 (15.2)	48 (35, 54)	
A5 < 40 mm		49 (33.6%)		
Amplitude at 10 mins (A10, mm)	147	52.9 (16.8)	59 (46, 64)	43–72
Maximum clot firmness (MCF, mm)	144	61.4 (15.6)	67 (58, 71)	56–78
Maximum Lysis (ML, %)	147	5.9 (5.7)	5 (2, 8)	0–14

^a^ Number of women with available data

^b^ Reference ranges for full blood count variables were obtained from the Sysmex KN analyser output. Reference ranges for thromboelastometry variables were obtained from literature [11]

Thirty five women (23%) had an EXTEM ML > 15%. If defined as an EXTEM A5 < 40 mm, coagulopathy was present in 49 (34%) mothers. If defined as a prothrombin ratio > 1.5, coagulopathy was present in 16 (12%) mothers. Of the women with an EXTEM A5 < 40 mm, 72% had a platelet count less than 100. The mean and median CT were markedly different suggesting that there are outliers. Of the 151 women with data for EXTEM CT, 127 women had a CT ≤ 100, 22 women had a CT between 101 and 1000 and two women had CTs of 1814 and 3468. If the two women with CT > 1000 are excluded, the mean (SD) and median (IQR) clotting times were 93.6 (138.7) and 54 (45, 70) respectively. The median (IQR) for plasma fibrinogen levels were 6.6 (3.2–12.3) g/L.

**Fig. 3:**
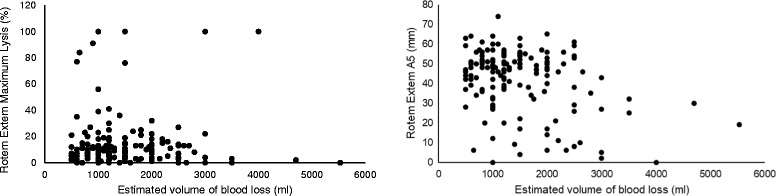
Scatterplot of the relationship between estimated blood loss and ROTEM ML and A5

Figure 3 shows the relationship between estimated blood loss and the coagulation parameters (maximal lysis and A5). There was no significant correlation between estimated blood loss and maximal lysis (*r* = 0.01, *p* = 0.86). There was a weak but statistically significant negative correlation between estimated blood loss and A5 (*r* = − 0.35, *p* < 0.001). After excluding women transferred from outside health facilities, the results were essentially the same (Maximal Lysis *r* = 0.06, *p* = 0.51; A5 *r* = − 0.46, *p* < 0.001).

## Discussion

Although 99% deaths from PPH occur in low and middle income countries, most of the research on the haemostatic abnormalities in PPH is from high income countries [13–15].

Among this cohort of women who received care at a Nigerian hospital for PPH, nearly one quarter of women had findings suggestive of hyper-fibrinolysis (ML > 15%). This contrasts with studies in high-income settings where hyper-fibrinolysis is considered less common in PPH [14] de Lange et al. reported that 9% of women after normal labour had ML of > 15% within 1 h of delivery of the placenta [12] with increased D-dimer levels. Older studies looking at peri-partum changes in fibrinolysis show increases in tissue plasminogen activator (t-PA) immediately after delivery, followed by increases in plasminogen activator inhibitor (PAI-1) [16, 17].

In patients with trauma, ROTEM appears to underestimate the prevalence of fibrinolytic activation compared with more sensitive measures such as plasmin-antiplasmin complexes [4]. Further studies are needed to examine which parameters are most sensitive in detecting fibrinolysis in obstetric bleeding. Although we did not find any correlation between estimated blood loss and fibrinolytic activity, inaccuracy in blood loss estimates could easily have obscured such an association. Indeed, evidence from the WOMAN trial that administration of tranexamic acid significantly reduces death due to bleeding and re-operation for bleeding, strongly suggests that fibrinolysis plays an important role in PPH. Furthermore, a randomised trial conducted in France has shown that there is an early increase in D-dimers and plasmin-antiplasmin complexes in women with active PPH and that this increase is attenuated among women who received tranexamic acid [18].

Fibrinogen levels would be expected to fall with increased fibrinolysis due to increased consumption and fibrinogenolysis. However, despite the high prevalence of hyper-fibrinolysis, the fibrinogen levels appeared to be elevated. Compared to non-pregnant women, fibrinogen levels are increased in pregnancy reaching their peak in the third trimester [19]. Nevertheless, the fibrinogen levels seen in our study are higher than in studies in high income settings. This might be related to the effects of inflammation due to acute and chronic infections including HIV and their treatments [20]. The prevalence of HIV in Nigeria is 3% and it is notable that 21% of our sample were lymphopenic. Further studies in low and middle income countries are need to confirm or refute our results.

Many women were anaemic and the degree of anaemia suggests that many were anaemic before developing PPH. Anaemia in Africa has multiple aetiologies including iron deficiency, functional iron deficiency (due infections such as malaria and HIV); genetic conditions such as sickle cell disease, thalassemia and glucose-6-phosphate dehydrogenase deficiency; parasitic infections leading to blood loss (e.g. hookworms); and drugs such as anti-retrovirals. Furthermore, due to the expansion of the plasma volume in pregnancy, haematocrit falls. Olatunbosun et al. found that 54% of women booking into an obstetric clinic in Uyo, Nigeria were anaemic, with most having evidence of iron deficiency [21]. Women with antenatal anaemia may have an increased risk of PPH and an increased risk of severe anaemia after delivery [22, 23].

One third of women was thrombocytopenic and many were severely thrombocytopenic. This also contrasts with studies in high income countries in which thrombocytopenia is relatively uncommon [24]. The low platelet counts (in the context of the relatively well preserved coagulation factors) may explain why so many women had an EXTEM A5 < 40 mm since EXTEM A5 values are influenced by platelet and fibrinogen levels. Further studies are needed to assess the FIBTEM and EXTEM A5 levels in similar populations to differentiate the extent to which low platelet and/or fibrinogen levels impact on PPH progression. Several studies have shown that a low A5 using ROTEM FIBTEM measured during the early phase of bleeding is associated with an increased risk of severe PPH [25].

Our study has several weaknesses. Because we did not collect blood samples prior to PPH onset, we cannot determine whether the abnormal haematological values (anaemia and thrombocytopenia) observed in this study are a cause or consequence of the bleeding. Due to technical problems with either blood samples or measurement instruments, the number of patients with available data was less than the number enrolled. We did not compare our results with a control group of women who did not experience PPH. Although the women in our study were a sample (54%) of all those recruited into the WOMAN trial at University College Hospital, we do not believe they were selected based on bleeding severity. The mean blood loss among the 167 women who were included was similar to that for the 142 women enrolled in the Woman trial but not included in this ROTEM study [1530 ml (SD) 897 for the included women versus 1548 (SD) 810] in women not included in the ROTEM study]. Their systolic blood pressures at baseline were also similar [110 mmHg (27) among included women versus 103 (33) in women who were not included]. It is possible that the reason for the high prevalence of coagulopathy found in our study is that our cohort had more severe PPH than studies in high income settings.

## Conclusions

Based on findings from a convenience sample of women who delivered in a teaching hospital in Nigeria, hyper-fibrinolysis occurs in nearly 25% of women with PPH. Further studies into the pathophysiological mechanisms for hyper-fibrinolysis should help us to identify better treatment strategies for women with PPH.

## Abbreviations

A5: Clot amplitude (firmness) at five minutes
aPTT: Activated partial thromboplastin time
EDTA: Ethylene diamine triacetic acid
ETAC: Effect of tranexamic acid on coagulation sub-study of woman trial.
EXTEM: Extrinsic screening test on ROTEM
g/L: Grams per litre
HIV: Human immunodeficiency virus
INTEM: Intrinsic screening test on ROTEM
L: Litre
LSHTM: London school of hygiene and tropical medicine
MCV: Mean corpuscular volume
ML: Maximum Lysis
mL: Millilitre
mmHg: Millimetre of mercury
mol/L: Moles per litre
NAFDAC: National agency for food & drug administration and control
PAI-1: Plasminogen activator inhibitors
PPH: Postpartum haemorrhage
PT: Prothrombin time
QC: Quality control
ROTEM: Rotational thromboelastometry
t-PA: Tissue plasminogen activator
WOMAN Trial: World maternal anti-fibrinolytic trial
